# Reappraising Out-of-Hospital Cardiac Arrest in Patients With Cancer

**DOI:** 10.1016/j.jacadv.2026.103380

**Published:** 2026-10-22

**Authors:** Alexi Vasbinder, Estelle Torbey, Richard K. Cheng

**Affiliations:** a Department of Biobehavioral Nursing and Health Informatics, University of Washington School of Nursing, Seattle, Washington, USA; b Division of Cardiology, Department of Internal Medicine, Brown University Health, Providence, Rhode Island, USA; c Division of Cardiology, Department of Medicine, University of Washington, Seattle, Washington, USA

**Keywords:** cancer, cardiac arrest, cardio-oncology, epidemiology, population-based

**Figure:**
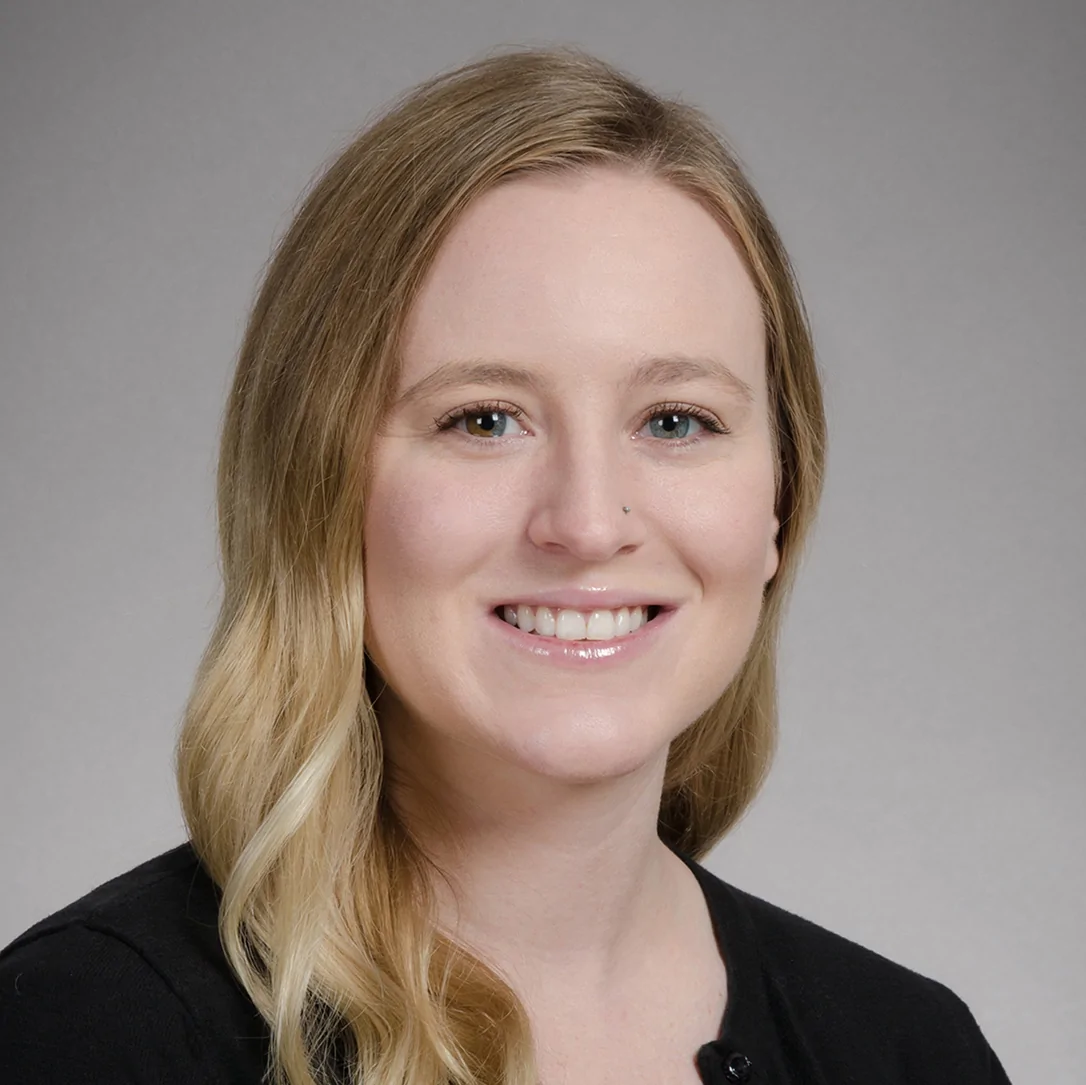

Cardiovascular disease is a leading cause of morbidity and mortality among patients with cancer. Shared risk factors, cancer-related inflammation and thrombosis, and cardiovascular toxicities of cancer therapy all contribute to this risk.^1^ However, out-of-hospital cardiac arrest (OHCA), one of the most consequential cardiovascular events, has received comparatively little attention in cardio-oncology. Prior studies have largely examined survival after OHCA among patients with cancer or described the clinical characteristics of those who arrest, drawing on cohorts that were regional in scope or underpowered to compare across cancer sites.^2^, ^3^ Whether cancer is independently associated with OHCA across cancer types and over time remains uncertain. This question is increasingly relevant with the growing burden of cancer survivors due to the aging population and improving outcomes in oncology.

In this issue of *JACC: Advances*, Hägglund et al^4^ leveraged linked Swedish national registries to address this question. The investigators included 798,451 adults diagnosed with cancer from 2010 through 2022 and 3,662,942 cancer-free controls matched on age, sex, region, and calendar period. OHCA was identified through the Swedish Register of Cardiopulmonary Resuscitation, which captures events for which cardiopulmonary resuscitation was initiated or continued. During a mean follow-up of 4.2 years among patients with cancer and 5.4 years among controls, cancer was associated with a 55% higher adjusted hazard of OHCA (HR: 1.55; 95% CI: 1.50-1.60). The excess hazard was greatest for lung cancer, which was associated with an almost 5-fold higher hazard (HR: 4.70; 95% CI: 4.36-5.08), followed by gastrointestinal and neurologic cancers, and was approximately 4-fold among patients with metastatic disease. Among individuals who experienced OHCA, patients with cancer had higher prevalence of heart failure, atrial fibrillation, other arrhythmias, and hypertension than cancer-free controls. The association between cancer and OHCA was stronger among younger adults, females, and individuals without baseline cardiovascular disease. Moreover, the association was strongest during the first year after cancer diagnosis but remained elevated beyond 2 years for several cancers, particularly lung cancer and multiple myeloma.

Particular strengths of the study include the national population-based design, large sample size, and linkage across 7 registries that enabled evaluation of OHCA across a broad spectrum of cancer sites. The availability of comorbidity, medication, socioeconomic, and mortality data supported adjusted and competing-risk analyses. Notably, the authors evaluated the strength of association by time since cancer diagnosis rather than estimating only an average association over follow-up. This distinction is particularly relevant in oncology, where cancer status, treatment exposure, complications, and prognosis evolve over time. The findings identify the first year after cancer diagnosis as a period of heightened OHCA occurrence. The variation across cancer sites is also informative. Melanoma and prostate cancer were not associated with OHCA, whereas lung cancer carried a nearly 5-fold higher hazard. Although residual confounding and differences in resuscitation practices may contribute to these estimates, this heterogeneity suggests that at least part of the excess hazard is related to specific cancers, their treatments, or associated complications rather than the cancer diagnosis alone.

At the same time, the excess risk identified in this study should not be assumed to be fully cardiac in origin. OHCA is a clinical endpoint with multiple potential causes. In registries, ventricular arrhythmia related to ischemia, cardiomyopathy, myocarditis, or QT prolongation may be indistinguishable from sudden arrest caused by pulmonary embolism, hemorrhage, sepsis, hypoxia, electrolyte abnormalities, or advanced cancer. Prior data suggest that noncardiac causes of arrest are more common among patients with cancer than among those without cancer.^3^ As not every case is adjudicated for etiology and comprehensive monitoring before and during the event is not always available for OHCA, attribution to cardiac vs noncardiac causes is often challenging. The strong associations observed with metastatic disease and with lung, gastrointestinal, and neurologic cancers therefore likely reflect several overlapping mechanisms, including baseline cardiovascular risk, cancer biology, treatment-related toxicities, and acute cancer–related complications.

Several methodological considerations are important. First, the outcome was resuscitation-attempted OHCA. The registry does not capture arrests in which resuscitation was not initiated because of a do-not-resuscitate order, a palliative setting, or definitive signs of death. This definition focuses on clinically actionable events, but it also incorporates decisions about prognosis, goals of care, and the appropriateness of resuscitation, creating the potential for selection bias. Patients with severe disease or poor clinical status may be less likely to receive attempted resuscitation and thus may be underrepresented, biasing the cohort toward a more favorable clinical profile. If patients were included and compared with cancer-free individuals of similar clinical severity, the observed association may have been attenuated because shared comorbidity and illness severity in both groups could dilute the excess risk attributable to cancer.

Second, detailed information on the presenting rhythm and adjudicated cause of arrest were not reported. Cancer treatment, recurrence, and progression were also not systematically captured. This limits the ability to determine whether the early increase in OHCA may be related to the cancer itself, acute illness surrounding diagnosis, perioperative events with surgery, or systemic cancer therapy. The approximately 6-fold early hazard in lung and neurologic cancers is difficult to interpret because it may reflect several mechanisms, including intensive treatment, pulmonary embolism, hypoxia, or bradyarrhythmia, rather than a single treatment effect. Unmeasured confounding from smoking, obesity, physical activity, and other environmental risk factors also remains possible despite adjustment for comorbidity and socioeconomic factors.

Third, relative and absolute risk provide different perspectives and need to be put into context clinically. The relative association between cancer and OHCA was stronger among younger adults, females, and individuals without baseline cardiovascular disease. However, these groups have lower background rates of OHCA and may not have the greatest absolute risk. In contrast, older adults and those with established cardiovascular disease may have a higher absolute burden even when the cancer-associated HR is smaller. The cumulative incidence of OHCA remained approximately 1% among females and adults younger than 65 years and approximately 2% among males and adults 65 years or older. Although the authors appropriately report both cause-specific and competing-risk estimates, the latter requires careful interpretation. A lower cumulative incidence in groups with high cancer mortality reflects that some patients die before OHCA can be observed. Death is not a random event, and patients who die early may also have a high underlying risk of OHCA. Thus, the lower cumulative incidence does not necessarily indicate lower susceptibility to OHCA; rather, it reflects the probability that OHCA will occur and be observed before death.

Ultimately, the clinical implications of these findings require careful framing. These results should not be interpreted as justification for routine ambulatory rhythm monitoring, advanced cardiac imaging, or dedicated cardio-oncology referral based solely on cancer site. Current cardio-oncology guidelines appropriately base surveillance intensity on a combination of cardiovascular risk, symptoms, and treatment exposure.^5^ Although certain cancers were associated with a higher relative risk of OHCA, the absolute event rates remained low, and the likelihood of death from nonarrhythmic causes often exceeded the likelihood of experiencing OHCA. Therefore, the clinical utility of identifying individuals at increased arrhythmic risk may be limited by competing mortality, particularly among patients with advanced malignancy.

Rather, the study underscores the need for careful cardiovascular assessment during the first year after cancer diagnosis, particularly among patients with high-risk cancer features or established cardiovascular disease. In clinical practice, this includes optimizing cardiovascular risk factors, recognizing and treating pre-existing cardiovascular disease, reviewing medications for QT-prolonging combinations, correcting electrolyte abnormalities, and promptly evaluating new chest pain, dyspnea, syncope, or palpitations. These actions are already recommended in clinical practice, but this study informs the relative timing.

The findings also have implications for shared decision-making. Survival after OHCA is poor in the general population and may be worse among patients with cancer.^6^ However, a cancer diagnosis alone is a poor guide to prognosis after arrest, which varies considerably by cancer site, stage, treatment response, functional status, and cause of arrest. Discussions about emergency planning and resuscitation preferences should therefore be individualized and revisited as the cancer trajectory changes. Population-level estimates can help anchor these conversations but should not be the sole determinant for an individual patient.

Further research is needed to connect this epidemiologic association to specific pathophysiologic mechanisms and potentially modifiable risk factors. Linking detailed cancer treatment data with presenting rhythm, presumed arrest etiology, location, bystander response, return of spontaneous circulation, and neurologic outcomes would help distinguish preventable cardiovascular events from arrests related to advanced illness. Treatment-specific and time-varying analyses are also needed to separate baseline susceptibility from acute and cumulative treatment effects. Before an OHCA risk model can be considered for clinical use, it must identify a group with sufficiently high absolute risk and lead to an intervention that improves outcomes while minimizing unnecessary testing burden.

In conclusion, Hägglund et al make an important contribution by showing that the association between cancer and OHCA varies by cancer site and by time since diagnosis. The next step is to determine how much of this excess risk is attributable to modifiable cardiovascular disease, cancer-related complications, or treatment exposure. Only then can this population-level association be translated into an effective prevention strategy.

## Funding support and author disclosures

Dr Vasbinder is supported by the 10.13039/100013382Institute of Translational Health Sciences, funded by the 10.13039/100006108National Center for Advancing Translational Sciences of the 10.13039/100000002National Institutes of Health under award number KL2TR002317. All other authors have reported that they have no relationships relevant to the contents of this paper to disclose.
